# Quantitative ESG disclosure and divergence of ESG ratings

**DOI:** 10.3389/fpsyg.2022.936798

**Published:** 2022-08-05

**Authors:** Min Liu

**Affiliations:** School of Business, Renmin University of China, Beijing, China

**Keywords:** ESG disclosure, ESG ratings, rating divergence, rating agency, quantitative disclosure

## Abstract

Over the past decade, sustainable finance has been a topic of burgeoning significance for investors, and ESG ratings have become commonly used to implement ESG investment strategies in practice. Strikingly, it is widely documented in both academic literature and investment practices that ESG ratings of a given firm can be extremely different across rating providers. However, despite the disagreement in ESG ratings being subject to a lot of criticism, only few studies have examined the sources and determinants of rating divergence. This study examines whether quantitative ESG disclosure is conducive to rating convergence among agencies. Based on ESG rating data of Chinese A-share listed companies, the author finds that greater quantitative ESG disclosure, especially disclosure on environmental and social pillars, results in greater divergence of ESG ratings. When employing a difference-in-differences design with a quasi-experiment of disclosure guidance introduced by Hong Kong Exchange, the results show that if ESG disclosure is standardized and comparable, more numerical information reduces agencies' rating disagreement instead. Further analyses show that the lack of agreement is related to a low rating in the future. The author also finds that the effect of quantified ESG disclosure on rating divergence is more pronounced when firms are single businesses rather than diversified businesses with poor ESG performance rather than good ESG performance. The results are robust to alternative measures of ESG rating divergence, alternative sample, two-way clustering, and additional control variables. Taken together, the results indicate that quantitative ESG disclosure degenerates rating disagreement.

## Introduction

In recent decades, companies have been under enormous pressure to be sustainable not only because of the obligation to create a favorable social impact for stakeholders other than shareholders but also because of the notion that environmental, social, and governance (ESG) issues are important for firms' competitiveness and legitimacy (Lins et al., 2017; Camilleri, 2018; Cao et al., 2019; Grewal and Serafeim, 2020). The 2020 Global Sustainable Investment Review (GSIR) reports the booming popularity of sustainable investments, with $ 35.3 trillion assets under management and 15% growth in 2 years. This implies that a remarkable demand for ESG ratings as a third-party assessment of ESG issues. Investors, firms, researchers, and even regulators rely on ESG rating agencies to evaluate firms' ESG performance. Ratings are regarded as a tool to address climate risks for institutional investors, equally as important as firm valuation models, shareholder proposals, and hedging (Krueger et al., 2020), and thus increasingly shape investment decisions (Hartzmark and Sussman, 2019). Moreover, an extensive stream of academic studies also draws conclusions from ESG ratings such as studies on economic consequences of ESG performance (Servaes and Tamayo, 2013; Cheng et al., 2014; Khan et al., 2016; Hubbard et al., 2017; Lins et al., 2017) and influence on asset pricing (Engle et al., 2020; Pedersen et al., 2021).

However, a lot of attention from investors, academic researchers, and the financial press has been drawn to the ESG rating disagreement among different rating agencies for the same firm (Chatterji et al., 2016; Gibson Brandon et al., 2021; Berg et al., 2022; Christensen et al., 2022). Taking the hot stock in Chinese A-share listed companies for example, Kweichow Moutai got a high “AA” from Sino-Securities Index but a low “C+” from SynTao Green Finance for its ESG performance by June 2020, which thoroughly confused investors. Regulators, including the United States SEC and the European Commission, have expressed a heightened concern on the quality and precision of ESG ratings. In general, the consistency of ESG ratings across different data providers reached an amazingly low correlation from 0.30 to 0.66 (Chatterji et al., 2016; Billio et al., 2021; Gibson Brandon et al., 2021; Jørgensen and Ellingsen, 2021). The lack of consistency calls into question the validity of ESG ratings, which could cause a whole set of adverse consequences as follows: first, the disagreement may potentially taint sustainable investment decisions, raising challenges for investors to integrate ESG dimensions into investment strategies and leading to inefficiencies in the capital market. Uncertainty emerging from ESG rating divergence commands an uncertainty premium to compensate for the additional exposure, as well as discourages ESG-sensitive investors' participation in the market (Gibson Brandon et al., 2021; Avramov et al., 2022). Second, rating disagreement could reduce incentives for firms to improve ESG performance, because there is no sense or commercial logic in spending substantial resources on activities that would result in obscure ratings. Third, the inconsistency of ESG ratings may shake the foundation of data analysis in academic research, resulting in inconsistency of conclusions. In short, it is crucial to establish a deeper understanding of the fact and reasons of ESG rating disagreement and explore how to improve the validity and convergence of ratings across rating agencies.

Recently, several studies have examined the sources and determinants of rating divergence. Chatterji et al. (2016) document a surprisingly low agreement of social ratings across six well-known information intermediaries and find that raters not only define CSR in various ways (theorization is low) but also use different methods and metrics to measure the same construct (commensurability is low). Furthermore, Berg et al. (2022) identify sources of ESG rating disagreement and decompose the divergence into three dimensions: using different categories (scope divergence), measuring the same categories with different indicators (measurement divergence), and taking different weights on the relative importance of the categories (weight divergence). They provide evidence that scope divergence and measurement divergence are the main drivers of rating divergence. More interestingly, Christensen et al. (2022) focus on how the extent of firms' ESG disclosure drives the agreement or disagreement of ESG ratings and find that greater disclosure leads to greater rating divergence, which is totally opposite to earnings forecasts in the equity markets and credit ratings in the debt market. The most important difference between these studies and the author's study is that they focus on explaining how the whole disclosure influences rating disagreement, while the author is interested in exploring whether quantitative ESG disclosure brings about some changes. On the one hand, consistent with ESG disclosure (Christensen et al., 2022), analysts may also have more opportunities for different interpretations of quantitative disclosure, giving rise to greater rating dispersion. On the other hand, quantitative disclosure not only markedly enhances the comprehensibility and comparability of ESG disclosure but also decreases analysts' subjectivity and discretion, thus setting stage for rating consensus. Hence, it is *ex ante* unknown whether numerical information would improve or deteriorate ESG rating convergence.

To shed light on this question, the author compiles ESG rating data from six ESG rating providers in China (SynTao Green Finance, Sino-Securities Index, China Alliance of Social Value Investment, WIND ESG, FTSE Russell, and Rankins), which represent the major players in Chinese ESG rating space. Given raters' coverage of public listed companies, the author restricts the sample to A-share firms listed on the Shanghai and Shenzhen stock exchanges in China and the sample period goes from 2014 to 2020, with 4,966 firm-year observations. Consistent with the average cross-correlations of ESG ratings from different agencies in prior studies (Gibson Brandon et al., 2021; Jørgensen and Ellingsen, 2021), most cross-correlations of ratings are between 0.4 and 0.6 in this study. After controlling for firm-level characteristics and some fixed effects, a preliminary regression analysis shows that greater quantitative ESG disclosure results in greater divergence of ESG ratings. Furthermore, the author disentangles the ESG disclosure and divided it into three pillars: E (the environmental pillar), S (the social pillar), and G (the governance pillar). The regression results are consistent with the conjecture of the author, who finds that disclosures on environmental and social issues contribute more to disagreement than governance disclosures.

To address the potential endogeneity problem and examine whether standardized quantitative disclosure helps to reduce the rating disagreement, the author conducts a quasi-experiment on the implementation of Environmental, Social, and Governance Reporting Guide introduced by Hong Kong Exchanges (HKEX) and conducts a difference-in-differences (hereafter DID) estimation. The results corroborate the main finding and suggest that consistent disclosure does not lead to disagreement, and that inconsistent disclosure does. Furthermore, the author explores the consequences of rating divergence and finds that the lack of agreement is associated with low rating in the future, implying that disagreement represents a source of uncertainty about potential risks. Cross-sectional analyses show that the effect of quantified ESG disclosure on rating disagreement is more significant when firms are single businesses rather than diversified businesses with poor ESG performance rather than good ESG performance. Lastly, the main results are robust to alternative measures of ESG rating divergence, alternative sample, two-way clustering, and additional control variables.

This study contributes to several streams of literature. First, it extends the literature on non-negligible divergence of ESG ratings among different rating agencies (Chatterji et al., 2016; Billio et al., 2021; Gibson Brandon et al., 2021; Jørgensen and Ellingsen, 2021). The findings of the author suggest that even quantitative ESG disclosure brings about multifarious interpretations of ESG performance and hence inconsistent ratings, which provide further empirical evidence for the presence and determinants of rating disagreement. This study is closely related to Christensen et al. (2022) and has a key conclusion that rating divergence is larger when firms disclose more. Specifically, this study differs from theirs in that the author places importance on how quantitative disclosure (i.e., numerical metrics) influences the evaluation process and results of information intermediaries about ESG issues. Second, this study complements research studies related to sociology of valuation and evaluation. Although quantification enhances comprehensibility and comparability by condensing information, restricting discretion, and simplifying decision-making (Porter, 1995; Espeland and Stevens, 1998, 2008), the author finds that rating disagreement is more pronounced when firms disclose more numerical information, indicating that low commensurability still poses a serious challenge to the convergence of ratings. In contrast, a shared cognitive system, including common definition and similar measurement of ESG performance (Sauder and Espeland, 2009; Hsu et al., 2012; Chatterji et al., 2016; Kotsantonis and Serafeim, 2019), plays a paramount role in driving convergence on social evaluation and judgment. Third, this study has implications for literature on economic consequences of ESG rating disagreement. Gibson Brandon et al. (2021) and Avramov et al. (2022) predict and find that firms with high ESG rating disagreement require an equity premium because disagreement is perceived as a source of uncertainty. This study complements some evidence and demonstrates that divergence is related to a lower average rating in the future, implying that quantitative disclosure degenerates rating disagreement but exposes potential ESG risks.

The reminder of the article proceeds as follows: Section Related research and hypothesis development provides a review of related research and develops testable hypotheses. Section Research design and data describes the research design and data. Section Results presents the main empirical results and interpretations. Sections Cross-sectional analyses and Robustness test report ion the cross-sectional analyses and robustness tests, respectively. Section Robustness test discusses the conclusions of this study in detail. Section Discussion concludes the article.

## Related research and hypothesis development

### Related research

#### ESG Ratings

Over the past decade, sustainable finance has been a topic of burgeoning significance for investors, and ESG ratings have become commonly used to implement ESG investment strategies in practice. In the meantime, a growing literature on management, economics, and finance also derives their conclusions from ESG ratings (Cheng et al., 2014; Khan et al., 2016; Avramov et al., 2022; Christensen et al., 2022). Most users are incapable of assessing the ESG performance of companies on their own and thus widely rely on ESG ratings from specialist rating agencies. The agencies, as information intermediaries, use a set of methods to identify risks and opportunities pertaining to ESG issues, transforming massive and complex information to aggregate scores (Escrig-Olmedo et al., 2019). ESG ratings support institutional investors with trillions of dollars in assets under management to integrate ESG dimensions into investment strategies and screen portfolios for risks and opportunities, which are previously unforeseen from financial performance (GSIR, 2020). Evidently, ESG ratings, like credit ratings in debt markets, can and do play a paramount role in capital allocation.

In China, there are several authoritative third-party data providers with expertise in evaluating ESG performance and localizing international scoring methodologies such as SynTao Green Finance, Sino-Securities Index, and China Alliance of Social Value Investment (CASVI). Rating agencies collect ESG information from various sources, assess it in their unique evaluation system, and produce ESG ratings or scores. Taking SynTao Green Finance for example, it collects self-disclosed information, such as annual reports and sustainability reports, and negative ESG information, such as formal media reports and penalties announced by regulatory authorities. The rating framework encompasses general indicators applicable to all companies and industry-specific indicators applicable to companies within industry classification (Broadstock et al., 2021). However, despite a great deal of time and energy spent on assessment, the validity and convergence of ESG ratings released by agencies are criticized in practice and research.

#### ESG rating disagreement

It has been debated critically in both academic literature and investment practices that the ESG ratings of a given firm can be extremely different across rating providers. Prior studies (Chatterji et al., 2016; Billio et al., 2021; Gibson Brandon et al., 2021; Jørgensen and Ellingsen, 2021) document a surprising lack of rating agreement between worldwide well-established information intermediaries, with an average correlation from 0.3 to 0.66. What is worse, Gibson Brandon et al. (2021) and Avramov et al. (2022) find that ESG rating disagreement seems to mislead even professional investors in their investment decisions and then discourages them from sustainable investment and active engagement in corporate ESG issues. The same applies to academics that draw plenty of influential conclusions. As such, it is crucial to explore why raters diverge widely; as a response, a growing number of studies have been dedicated to this topic.

Researchers have found some theoretical underpinnings in the literature related to sociology of valuation and evaluation. There are two prerequisites to converge on assessments for raters: theorization and commensurability (Durand et al., 2007; Espeland and Sauder, 2007; Sauder and Espeland, 2009; Hsu et al., 2012). A common theorization implies convergence on a common definition of ESG across ESG rating agencies. Despite a broad rhetorical agreement on the components of ESG performance, there are actually dramatic differences in the way raters theorize ESG, and high-level scope divergence (e.g., different sets of attributes between the scope of ratings) is one of the main drivers of rating divergence (Chatterji et al., 2016; Berg et al., 2022). However, after adjusting for differences in theorization, rating divergence may remain high as a result of low commensurability. High commensurability means that raters employ similar measurements and interpretations of the same construct. In practice, different raters generally measure the same attribute with various indicators, which make it impracticable to compare across ESG ratings and hence lead to great disagreement. Berg et al. (2022) provide convincing empirical evidence that measurement divergence is the most important force of rating divergence based on the data set of ESG ratings from six prominent agencies.

## Hypothesis development

Drawing on studies on investor disagreement in financial markets, two main sources of disagreement are differences in information sets and differences in interpretations of information (Hong and Stein, 2007; Cookson and Niessner, 2020). Financial disclosures usually reduce dispersion among equity analysts, as well as credit rating analysts owing to widespread agreement on the meaning of financial information (Morgan, 2002; Hope, 2003; Bonsall and Miller, 2017; Akins, 2018), while ESG disclosures are likely to exacerbate dispersion among ESG rating analysts because of little agreement on how to interpret and judge the meaning of ESG performance disclosures. According to this viewpoint, Christensen et al. (2022) find empirical evidence that greater ESG disclosure gives rise to greater ESG rating disagreement. Nevertheless, what about quantitative ESG disclosure?

On the one hand, greater quantitative ESG disclosure may lead to greater ESG rating divergence. In the ideal situation, if rating agencies adopt the same approach to evaluate firms' ESG performance, the consistency of ratings should be fairly high no matter how much firms disclose. Nonetheless, the fact is that there are thousands of ways that firms report their ESG data and raters make their assessments, with different terminologies and different units of measure. To illustrate, Kotsantonis and Serafeim (2019) selected a random sample of 50 listed firms in Fortune 500 and found that the 50 firms use more than 20 distinct metrics to report on the issue of employee health and safety, e.g., lost time frequency rate, total case incident rate, number of severe accidents that occurred, occupational disease rate, and number of lost workdays. These indicators are similar but not identical and numerical but incomparable. Evidently, there is no consensus on which of these metrics best capture good ESG performance and how they are aggregated into an assessment system (Chatterji et al., 2016; Christensen et al., 2022). For firms with low level of quantitative ESG disclosure, agencies commonly have little dispute about their performance ranking near the bottom, whereas for firms disclosing many numerical information, things begin to get cluttered. Raters need to interpret and judge whether figures present good or bad ESG performance and compare the figures with those of peer firms despite incommensurability of different metrics. Accordingly, the author formulates the following hypothesis:

H1a: quantitative ESG disclosure is positively related to the divergence of ESG ratings.

On the other hand, greater quantitative ESG disclosure may bring about lower ESG rating divergence. In many areas of social sciences, the logic of quantification is implicit in a broad range of valuation/evaluation systems^1^. Quantification transforms quality into quantity and difference into numbers, integrating information into a shared cognitive system (Espeland and Stevens, 2008). The dominant reason why quantitative ESG disclosure may mitigate rating disagreement is that by condensing information and simplifying decision-making, quantification markedly enhances the comprehensibility and comparability of ESG disclosure (Espeland and Stevens, 1998). Compared with “establish a timely and quick consumer response system oriented to consumer needs”, it is apparently easier to assure consistency and reach consensus on “customer satisfaction is 95%” for rating agencies. In addition, numerical information, characterized as mechanical objectivity, restricts discretion especially when the credibility is challenged (Porter, 1995; Espeland and Stevens, 2008). Tang et al. (2021) find that companies held by the same owners as the rating agency receive higher ESG ratings, suggesting that the conflicts of interest degenerate the quality of ESG ratings. Hence, quantitative ESG disclosure is so impersonal and comparable that raters would decrease discretion and make consistent judgments. Collectively, the author proposes the following alternative hypothesis:

H1b: quantitative ESG disclosure is negatively related to the divergence of ESG ratings.

Whether quantitative ESG disclosure alleviates or exacerbates rating divergence is unknown. However, when it comes to standardized quantitative disclosure, the situation has become relatively clear. If companies disclose their ESG activities and performances in standardized and comparable indicators, rating agencies are more likely to absorb the commonly used indicators into their evaluation systems and make horizontal comparisons between companies. Standardized quantitative disclosure, following sustainable reporting instruments and standards developed by regulators or non-governmental organizations, is conducive to the rating convergence among agencies for the following reasons: (1) rating agencies tend to incorporate the indicators required by reporting instruments or standards into their assessment process, which greatly narrows the differences between agencies' evaluation systems and then converge rating outcomes; (2) when horizontal comparisons between companies are easily implementable, different rating agencies would choose similar companies' portfolio as highly rated companies.

To examine this conjecture, I use the *Environmental, Social and Governance Reporting Guide* introduced by Hong Kong Exchanges (HKEX) as a setting of improvement of ESG data consistency. Similar to government-initiated policies on ESG in the European context (Camilleri, 2015), this Guide requires a series of standardized key performance indicators, and firms must report on the “comply or explain” provisions of this Guide. Obviously, the new guidance greatly elevates the formalization and comparability of ESG reports. Although there are no explicit regulations for A-share firms listed on Shanghai and Shenzhen stock exchanges, a small part of these firms is cross-listed on HKEX. From July 2020 onward, companies cross-listed on A-shares and HKEX have to disclose standardized and comparable ESG performance measures following the guidance of HKEX, while companies only listed on A-shares are not subject to it. The author predicts that firms cross-listed on HKEX disclose more standardized quantitative indicators and get more consistent ESG ratings from agencies. In other words, agencies are more likely to reach a consensus about standard indicators disclosed by firms who have to meet the mandatory disclosure requirements. Therefore, the author proposes the second hypothesis:

H2: Standardized quantitative ESG disclosure is negatively related to the divergence of ESG ratings.

## Research design and data

### Research design

To test the hypothesis that greater quantitative metrics of ESG disclosure will give rise to greater divergence of ESG ratings, the main identification model is as follows:


(1) $$\begin{array}{l} ESG\_Divergence_{i,t}\;=\;a_{0}\;+\;a_{1}ESG\_Qmetrics_{i,t}\\ \;+\text{​}\sum_{\;}^{\;}a_{k}Control_{i,t}+\;FixedEffects\;+\;\varepsilon_{i,t} \end{array}$$

where *i* indexes firm, *t* indexes year. *ESG_Divergence*_*i, t*_ is the standard deviation of ESG ratings of firm *i* in year *t*, and *ESG_Qmetrics*_*i, t*_ is the natural logarithm of the number of quantified indicators about ESG disclosure plus one. The construction of the two variables will be explained in detail later. The author predicts that the coefficient of interest α_1_ will be significantly positive.

*Control*_*i, t*_ contains a vector of firm-level control variables, including the average ESG rating of a firm received from different rating agencies (*ESG_mean*), the number of rating agencies following the firm (*ESG_N*), state-owned enterprise or not (*State*), total assets (*Size*), capital structure (*Leverage*), profitability (*ROA*), book-to-market ratio (*BM*), and Tobin's Q (*TobinQ*) (refer to detailed definitions in Appendix A). Fixed effects consist of firm, year, and rating agency. For the robustness of the results, the author uses industry, year, and rating agency fixed effects. Standard errors are clustered at the firm level.

Next, to examine whether standardized quantitative disclosure mitigates rating disagreement (*H2*), the author constructs the following DID model, taking *Environmental, Social and Governance Reporting Guide* introduced by Hong Kong Exchanges as an exogenous shock:


(2) $$\begin{array}{l} ESG\_Divergence_{i,t}\;=\;\gamma_{0}\;+\;\gamma_{1}ESGQmetrics_{i,t}\;\times \;D_{i,t}\\ \;+\;\gamma_{2}ESGQmetrics_{i,t}\;+\;\gamma_{3}D_{i,t}\\ \;+\text{​}\sum_{\;}^{\;}\gamma_{k}Control_{i,t}+\;FixedEffects\;+\;\tau_{i,t} \end{array}$$

where *D*_*i, t*_ is a dummy variable equal to 1 for A-share companies cross-listed on HKEX after July 2020 and 0 otherwise. *ESG_Divergence*_*i, t*_, *ESG_Qmetrics*_*i, t*_, *Control*_*i, t*_, and fixed effects are the same as in Equation (1). γ_1_ is the coefficient of interest, and it is predicted to be significantly negative.

### Data

The common challenge faced by ESG research is that data points of ESG ratings are few in both the cross-section and time series, and access to these data has not been sufficient. To include as many raters as possible, the author uses Chinese ESG rating data provided by six ESG rating agencies: SynTao Green Finance, Sino-Securities Index, CASVI, WIND ESG, FTSE Russell, and Rankins. Most are available through the WIND database, and a few are extracted from annual ESG rating reports. Together, these agencies represent the major players in Chinese ESG rating space, which cover a substantial part of the ESG ratings market.

As Table 1 shows, these agencies have different frequencies in releasing updated ratings: Sino-Securities Index and WIND ESG are quarterly, CASVI is semi-yearly, and others are yearly. Because of the annual ratings of some agencies as well as rare varieties of ratings within a year, the author bases the scores on firm-year level. According to the information disclosure requirement of China Securities Regulatory Commission (CSRC), Chinese A-share listed companies generally publish the prior year's annual financial report and CSR/ESG report in the first half of year. Therefore, if an agency issues more than one rating for a specific firm-year observation, the author keeps the rating that is released nearest to the middle of the year. Moreover, to make different ratings comparable, the author rescales the rating score, ranging from 0 to 10. Based on the standardized data, variable *ESG_Divergence* is defined as the standard deviation of ESG ratings of a firm-year from different agencies.

**Table 1:** Rating overview using available information in the database.

**Rating agency**	**Rating score**	**Coverage**	**Frequency**	**Source**
SynTao Green Finance	D to A+	HuShen 300 (2014–2020), CSI 500 (2018–2020)	Yearly	WIND database
Sino-Securities Index	C to AAA	A-share companies (2009–2020)	Quarterly	WIND database
CASVI	D to AAA	HuShen 300 (2015–2020)	Semiyearly	WIND database
WIND ESG	0 to 10	CSI 800 (2016–2020)	Quarterly	WIND database
FTSE Russell	0 to 5	(2017–2020)	Yearly	WIND database
Rankins	CCC to AAA	CSI 800 (2019–2020)	Yearly	Annual ESG rating reports

The number of quantified indicators about firms' ESG disclosure is provided by China Stock Market & Accounting Research Database (CSMAR). CSMAR extracts substantial and quantified metrics related to environmental, social, and governance activities and performances by scanning through firms' CSR/ESG reports. Depending on these data points, variable *ESG_Qmetrics* counts the number of quantified indicators about firms' ESG disclosure. The firm characteristic data used in regressions is also from the CSMAR database.

The author restricts the sample to A-share firms listed on the Shanghai and Shenzhen stock exchanges in China, and the sample period goes from 2014 to 2020. To meet the requirement of research design, the author excludes: (1) firm-years with <2 ESG ratings, (2) finance companies, and (3) firm-years with missing variables. The final sample includes 1,024 companies in China and 4,966 firm-year observations. All continuous variables are winsorized at the upper and lower 2% levels, avoiding the impact of extreme outliers.

### Descriptive statistics

Table 2 Panel A reports summary statistics and Pearson correlations between ESG ratings from each rating agencies in the sample. After standardization ranging from 0 to 10, Sino-Securities Index tends to issue high ESG scores (average of 7.846), while FTSE Russell and Rankins tend to issue much lower scores (average of 2.46 and 2.652). In view of the different scoring tendencies of these ESG rating agencies, it is necessary to control rating agencies' fixed effects in subsequent estimations. The variance across firms of CASVI scores and Sino-Securities Index scores is relatively high, 1.438 and 1.426 specifically. It is worth noting that the mean score issued by SynTao Green Finance is around 5 (5.19) and that the variance across firms (standard deviation of 0.981) is least among the six agencies. The right side of Table 2 Panel A shows the cross-correlations of ESG ratings from different agencies. All these relationships are significant at 1% level. Consistent with the average correlation of each respective cross-correlation in Gibson Brandon et al. (2021) and Jørgensen and Ellingsen (2021) (0.447 and 0.664 respectively), most Pearson correlations of agency scores are between 0.4 and 0.6. The strongest relationship is the pairwise correlation between WIND ESG and Rankins scores (0.668), while the lowest correlation is between SynTao Green Finance and Sino-Securities Index.

**Table 2:** Descriptive statistics.

**Panel A: Descriptive statistics and correlations for ESG ratings from each rating agency**									
**ESG ratings from agencies**	**N**	**Mean**	**Median**	**Standard deviation**	**Pearson correlations**				
					**SynTao Green Finance**	**Sino-Securities Index**	**CASVI**	**WIND ESG**	**FTSE Russell**
SynTao Green Finance	4,277	5.190	5.000	0.981					
Sino-Securities Index	4,966	7.846	7.780	1.426	**0.262**				
CASVI	1,972	6.523	6.625	1.438	**0.404**	**0.372**			
WIND ESG	3,252	6.770	6.660	1.059	**0.577**	**0.399**	**0.520**		
FTSE Russell	1,905	2.460	2.200	1.036	**0.608**	**0.284**	**0.458**	**0.569**	
Rankins	1,288	2.652	2.860	1.276	**0.595**	**0.544**	**0.591**	**0.668**	**0.581**
**Panel B: Descriptive statistics of regression variables**						
**Variables**	**N**	**Mean**	**Standard Deviation**	**Percentile 25**	**Median**	**Percentile 75**
*ESG_Divergence*	4,966	2.051	0.862	1.404	2.073	2.635
*ESG_Qmetrics*	4,966	0.847	1.014	0.000	0.000	1.609
*ESG_mean*	4,966	5.966	1.040	5.250	5.984	6.737
*ESG_N*	4,966	3.556	1.342	2	3	5
*State*	4,966	0.482	0.500	0	0	1
*Size*	4,966	23.690	1.234	22.800	23.550	24.440
*Leverage*	4,966	0.474	0.193	0.324	0.485	0.622
*ROA*	4,966	0.055	0.064	0.019	0.044	0.086
*BM*	4,966	0.681	0.283	0.458	0.693	0.919
*TobinQ*	4,966	1.936	1.350	1.088	1.443	2.182
**Panel C: Correlations of regression variables**										
	***ESG_Divergence***	***ESG_Qmetrics***	***ESG_mean***	***ESG_N***	***State***	***Size***	***Leverage***	***ROA***	***BM***	***TobinQ***
*ESG_Divergence*	1									
*ESG_Qmetrics*	**0.161**	1								
*ESG_mean*	**−0.221**	**0.513**	1							
*ESG_N*	**0.258**	**0.244**	**−0.171**	1						
*State*	**0.136**	**0.325**	**0.255**	**0.066**	1					
*Size*	**0.182**	**0.432**	**0.236**	**0.378**	**0.395**	1				
*Leverage*	**0.053**	**0.172**	**0.062**	**0.043**	**0.196**	**0.593**	1			
*ROA*	0.022	**−0.053**	**0.099**	**0.097**	**−0.224**	**−0.225**	**−0.479**	1		
*BM*	**0.101**	**0.207**	0.011	**0.045**	**0.315**	**0.626**	**0.507**	**−0.505**	1	
*TobinQ*	−0.025	**−0.149**	**−0.032**	**0.05**	**−0.315**	**−0.471**	**−0.439**	**0.505**	**−0.836**	1

*This table presents descriptive statistics of the sample. Panel A reports summary statistics and Pearson correlations of ESG ratings from each agency. Panel B shows descriptive statistics of regression variables. Panel C presents Pearson correlations of regression variables. Bold numbers in Panel A and Panel C denote Pearson correlations significant at the 5% level. See Appendix A for detailed definitions of the variables*.

Table 2 Panel B displays the summary statistics of the variables used in the main analyses. The mean *ESG_Qmetrics* is 0.847, meaning that firms disclose approximately 0.847 (natural logarithm) substantial and quantified metrics related to ESG performance. These firm-years are followed by 3.556 ESG rating agencies and get a score of 5.966 on average. In this sample, an average firm has a leverage of 47.4%, size of 23.69, ROA of 0.055, book-to-market ratio of 0.681, and Tobin's Q of 1.936. Besides, a total of 48.2% of firms are state-owned enterprises.

Table 2 Panel C shows the Pearson correlation coefficients of the variables. *ESG_Qmetrics, ESG_mean* and *ESG_N* are positively correlated with *State* and *Size*, which suggests that state-owned enterprises and fairly large firms disclose more quantitative information about ESG performance, attract more agencies, and get higher ESG ratings. *ESG_N* is positively correlated with *ESG_Divergence*, while *ESG_mean* is negatively correlated with *ESG_Divergence*. The correlation between *ESG_Qmetrics* and *ESG_Divergence* is 0.161, which provides preliminary evidence for the hypothesis of the author.

## Results

### ESG numerical disclosure and ESG ratings divergence

Table 3 shows the regression results of Equation (1) using different specifications. Column (1) reports the result of a regression with observable firm-level characteristics, column (2) provides the result controlling for the same characteristics as well as ESG rating agency, industry, and year fixed effects, while column (3) includes firm characteristics and fixed effects of ESG rating agency, firm, and year to control for unobservable factors in agency-, firm- and year-levels. The coefficient of *ESG_Qmetrics* is the coefficient of interest. The point estimates in columns (1) to (3) are 0.242, 0.108, and 0.061 respectively, statistically significant at the 1% level. Given that the sample standard deviation of *ESG_Divergence* is 0.862, the economic magnitude of the effect is substantial. Overall, these findings corroborate H1a that if a firm discloses more non-standardized numerical information about its ESG performance, it will receive more divergent ESG scores from rating agencies. Quantified indicators in ESG disclosure pose a great challenge for rating agencies to assess the performance and compare with peers.

**Table 3:** Effect of ESG quantitative disclosure on ESG rating divergence.

**Dependent variable**	**(1)**	**(2)**	**(3)**
	***ESG_Divergence***	***ESG_Divergence***	***ESG_Divergence***
***ESG_Qmetrics***	0.242***	0.108***	0.061***
	**(10.58)**	**(5.05)**	**(3.10)**
*ESG_mean*	−0.361***	−0.088***	−0.063*
	(-14.05)	(−3.27)	(−1.82)
*ESG_N*	0.024	−0.115**	0.393***
	(1.38)	(−2.01)	(7.50)
*State*	0.215***	0.216***	0.116
	(5.23)	(5.83)	(1.17)
*Size*	0.102***	0.085***	0.159***
	(4.02)	(3.49)	(3.36)
*Leverage*	−0.067	−0.227**	−0.336**
	(−0.53)	(−2.05)	(−2.06)
*BM*	0.071	−0.199*	−0.289***
	(0.62)	(−1.93)	(−2.71)
*TobinQ*	0.038*	0.002	−0.004
	(1.95)	(0.11)	(−0.21)
*ROA*	1.453***	1.212***	0.365
	(4.81)	(4.65)	(1.36)
Constant	1.221**	0.888	−1.421
	(2.47)	(1.64)	(−1.40)
Year FE	No	Yes	Yes
Agency FE	No	Yes	Yes
Industry FE	No	Yes	No
Firm FE	No	No	Yes
Observations	4,966	4,966	4,966
R^2^	0.198	0.467	0.487

This table presents the regression results of Equation (1) using different specifications. The dependent variable ESG_Divergence is the standard deviation of ESG ratings. In column (1), the author regresses ESG_Divergence on ESG_Qmetrics, and firm-level control variables. In column (2), the author adds agency, industry, and year fixed effects. In column (3), the author replaces industry fixed effects with firm fixed effects. Definitions of the variables are provided in Appendix A. The standard errors are clustered at the firm level. Values of t-statistics are in parentheses. Statistical significance at the 1, 5, and 10% levels is indicated by ***, **, and *, respectively. Bold text denotes the key variables and their estimated coefficients.

With regard to control variables, the coefficient of *ESG_mean* is negative and statistically significant across all the model specifications reported in Table 3, suggesting that ESG rating agencies tend to split on firms with poor ESG performance. The estimation coefficient on *ESG_N* is significantly positive in column (3) (coefficient = 0.393, *t* = 7.5), which is consistent with the intuition and the Pearson correlation test. When more ratings are available, the disagreement tends to be more considerable. In addition, two firm characteristics play an obvious role in explaining ESG ratings dispersion: the point estimates of *State* and *Size* are positive and almost significant. State-owned firms exhibit higher ESG rating disagreement than non-state-owned firms because they usually take social responsibility associated with national strategic goals such as targeted poverty alleviation and employment creation, and agencies may allocate different weights in these aspects. Also, ESG activities in larger firms are personalized, and it is hard to find comparable companies, which may lead to radically different ratings from agencies.

Furthermore, the author examines whether disclosure for some pillars (E, S, and G) contributes more to rating disagreement. Specifically, environmental and social issues have been debated for a shorter period than governance, and there is less of a general consensus on environmental and social pillars (Christensen et al., 2022). Based on the previous discussion, the lack of common understanding of the definition of ESG, including what are good performances and how to measure them, plays an essential role in explaining rating divergence. Therefore, the author expects that disclosures for environmental and social pillars should be more likely to result in rating divergence.

The author disentangles ESG disclosure and divided it into three pillars: E (the environmental pillar), S (the social pillar), and G (the governance pillar). The author then re-estimates Equation (1) and replace *ESG_Qmetrics* with *ESG_Qmetrics_E, ESG_Qmetrics_S*, and *ESG_Qmetrics_G* separately. Table 4 shows the regression results. As expected, after controlling for firm characteristic variables and year-, agency- and firm-level fixed effects, the coefficients on *ESG_Qmetrics_E* and *ESG_Qmetrics_S* are significantly positive, whereas the coefficient on *ESG_Qmetrics_G* is insignificant. These findings imply that disclosures on environmental and social issues contribute more to ESG rating divergence.

**Table 4:** Effect of E/S/G pillar-specific quantitative disclosure on ESG ratings divergence.

**Dependent variable**	**(1)**	**(2)**	**(3)**
	***ESG_Divergence***	***ESG_Divergence***	***ESG_Divergence***
***ESG_Qmetrics_E***	0.058*		
	(1.96)		
***ESG_Qmetrics_S***		0.057**	
		(2.51)	
***ESG_Qmetrics_G***			0.048
			(1.06)
*ESG_mean*	−0.033	−0.048*	−0.022
	(−1.43)	(−1.87)	(−1.00)
*ESG_N*	−0.093	−0.099*	−0.090
	(−1.62)	(−1.72)	(−1.56)
*State*	0.230***	0.222***	0.231***
	(6.19)	(5.96)	(6.22)
*Size*	0.092***	0.091***	0.094***
	(3.71)	(3.68)	(3.80)
*Leverage*	−0.218*	−0.224**	−0.219**
	(−1.95)	(−2.02)	(−1.97)
*BM*	−0.203*	−0.193*	−0.201*
	(−1.93)	(−1.85)	(−1.91)
*TobinQ*	0.001	0.001	0.000
	(0.04)	(0.07)	(0.02)
*ROA*	1.120***	1.145***	1.102***
	(4.27)	(4.38)	(4.22)
Constant	0.197	0.321	0.086
	(0.40)	(0.65)	(0.17)
Year FE	Yes	Yes	Yes
Agency FE	Yes	Yes	Yes
Industry FE	Yes	Yes	Yes
Observations	4,966	4,966	4,966
R^2^	0.452	0.453	0.451

This table presents the regression results of the effect of environmental pillar, social pillar, and governance pillar disclosure on rating disagreement. The author re-estimates Equation (1) and uses ESG_Qmetrics_E, ESG_Qmetrics_S, and ESG_Qmetrics_G as the key independent variables in columns (1) to (3), respectively. ESG_Qmetrics_E is the natural logarithm of the number of quantified indicators for environmental pillar about firm i's ESG disclosure plus one. ESG_Qmetrics_S is the natural logarithm of the number of quantified indicators for social pillar about firm i's ESG disclosure plus one. ESG_Qmetrics_G is the natural logarithm of the number of quantified indicators for governance pillar about firm i's ESG disclosure plus one. Firm-level characteristic variables, as well as agency, industry, and year fixed effects are controlled. The definitions of the variables are provided in Appendix A. The standard errors are clustered at the firm level. The values of t-statistics are in parentheses. Statistical significance at the 1, 5, and 10% levels is indicated by ***, **, and *, respectively. Bold text denotes the key variables and their estimated coefficients.

### Standardized quantitative ESG disclosure and ESG ratings divergence

Section ESG numerical disclosure and ESG ratings divergence shows that the more non-standardized numerical information about ESG performance a firm discloses, the more divergent ESG ratings it gets from agencies. In this section, the author explores what could happen if companies disclose quantitative indicators following the reporting standard.

The underlying argument of the main conclusion is that multifarious quantified disclosure provides rating agencies with more information to interpret and evaluate the performance, which leads to a different judgment. For example, firm A's number of accidents with fatal consequences is 1 and firm B's injury rate is 5%. Since the two metrics are not necessarily the same thing, agency C may choose firm A as a better performer on health and safety, while agency D may choose the other. Besides, agency C probably chooses the number of accidents with fatal consequences as one of the key indicators to evaluate firms' ESG performance, while agency D does not. In this case, as mentioned in *H2*, standard ESG data will narrow this kind of differences markedly.

Table 5 reports the regression results of a DID estimation using Equation (2). As shown in columns (1) and (2), the point estimates of *ESG_Qmetrics* are positive and significant at the 1% level, consistent with the main results. Moreover, the coefficient on the interaction term ESG_Qmetrics^*^D is significantly negative, which indicates that if ESG disclosure is standardized and comparable, more numerical information reduces agencies' rating disagreement instead. The results suggest that due to the inconsistency of disclosure, more quantitative metrics of ESG disclosure exacerbate disagreement across ESG rating agencies.

**Table 5:** Effect of standardized quantitative ESG disclosure on ratings divergence: using the shock of disclosure guidance in HKEX.

**Dependent variable**	**(1)**		**(2)**	
	***ESG_Divergence***		***ESG_Divergence***	
	**Coefficient**	***t*-statistic**	**Coefficient**	***t*-statistic**
ESG_Qmetrics*D	−0.156***	**(−2.62)**	−0.137**	**(−2.02)**
***ESG_Qmetrics***	0.092***	**(5.02)**	0.065***	**(3.32)**
*D*	0.088	(0.57)	0.172	(0.98)
*ESG_mean*	−0.081***	(−2.94)	−0.060*	(−1.73)
*ESG_N*	0.119**	(2.51)	0.392***	(7.50)
*State*	0.190***	(4.98)	0.110	(1.11)
*Size*	0.129***	(5.24)	0.155***	(3.27)
*Leverage*	−0.319***	(−2.91)	−0.344**	(−2.10)
*BM*	−0.330***	(−3.51)	−0.291***	(−2.74)
*TobinQ*	−0.003	(−0.18)	−0.005	(−0.28)
*ROA*	0.925***	(3.79)	0.370	(1.38)
Constant	−0.524	(−0.96)	−1.338	(−1.32)
Year FE	Yes	Yes
Agency FE	Yes	Yes
Industry FE	Yes	No
Firm FE	No	Yes
Observations	4,966	4,966
R^2^	0.476	0.488

This table presents the regression results of the mechanism. The dependent variable ESG_Divergence is the standard deviation of ESG ratings. Based on Equation (2), the estimations include ESG_Qmetrics, D, and the interaction term ESG_Qmetrics^*^D. D is equal to 1 for cross-listed companies on A-shares and HKEX after July 2020 and 0 otherwise. In column (1), the author controls for firm-level variables, as well as year, agency, and industry fixed effects. In column (2), the author replaces industry fixed effects with firm fixed effects. The definitions of the variables are provided in Appendix A. The standard errors are clustered at the firm level. Statistical significance at the 1, 5, and 10% levels is indicated by ***, **, and *, respectively. Bold text denotes the key variables and their estimated coefficients.

### Consequences: ESG rating in the future

Gibson Brandon et al. (2021) and Avramov et al. (2022) find that ESG rating disagreement is positively associated with cost of equity capital. They conjecture that higher ESG rating disagreement is perceived as a source of uncertainty, which requires an uncertainty premium. In this section, the author examines whether ESG performance in the future would embody the uncertainty risks implied by rating disagreement. There are two possible circumstances to account for the lack of consensus on a firm's ESG rating: a positive ESG activity is highly rated by an agency and undervalued by another or a negative ESG activity is identified as an insignificant thing by an agency and a warning sign by another. Material and sustainable ESG activities do not usually lead to disagreement, while ESG issues with potential risks are more likely to bring about different interpretations from rating agencies. If potential risks do exist, firms with ESG rating disagreement will perform worse in the following years on average. To test this conjecture, the author constructs Equation (3) as follows:


(3) $$\begin{array}{l} ESG\_Mean_{i,t\;+\;1}\;=\;\beta_{0}\;+\;\beta_{1}ESG\_Divergence_{i,t}\\ \;+\text{​​​​}\sum_{\;}^{\;}\beta_{k}Control_{i,t}\;+\;FixedEffects\;+\;\mu_{i,t} \end{array}$$

The dependent variable is *ESG_mean* for firm *i* in year *t* + *1*, and the independent variable is *ESG_Divergence* for firm *i* in year *t*. Controls contain a vector of firm characteristics, including *ESG_N, ESG_Qmetrics, State, Size, Leverage, BM, TobinQ*, and *ROA* (refer to detailed definitions in Appendix A). Table 6 shows the regression results using two specifications: (1) with year, agency, and industry fixed effects and (2) with year, agency, and firm fixed effects. The negative coefficients of *ESG_Divergence* in columns (1) and (2) indicate that firms with great ESG rating divergence will experience considerably lower ESG ratings in the future, providing further evidence of risk premium being related to ESG uncertainty.

**Table 6:** Additional analyses: the consequence of ESG rating divergence on future ratings.

**Dependent variable**	**(1)**		**(2)**	
	***F.ESG_mean***		***F.ESG_mean***	
	**Coefficient**	***t*-statistic**	**Coefficient**	***t*-statistic**
***ESG_Divergence***	−0.078***	**(−3.26)**	−0.041*	**(−1.87)**
*ESG_N*	−0.291***	(−5.91)	−0.210***	(−4.61)
*ESG_Qmetrics*	0.472***	(24.25)	0.145***	(6.94)
*State*	0.173***	(4.16)	−0.116	(−0.91)
*Size*	0.067**	(2.22)	−0.085	(−1.64)
*Leverage*	−0.123	(−0.93)	0.064	(0.37)
*BM*	−0.285**	(−2.16)	−0.161	(−1.25)
*TobinQ*	−0.038*	(−1.74)	−0.035	(−1.64)
*ROA*	1.432***	(4.74)	0.661**	(2.36)
Constant	4.726***	(6.52)	8.797***	(7.38)
Year FE	Yes	Yes
Agency FE	Yes	Yes
Industry FE	Yes	No
Firm FE	No	Yes
Observations	3,912	3,912
R2	0.577	0.480

This table presents the regression results of the consequences. The dependent variable ESG_mean in the next period is the average ESG rating of a firm received from different rating agencies. The independent variable ESG_Divergence is the standard deviation of ESG ratings. In column (1), the author controls for firm-level variables, as well as year, agency, and industry fixed effects. In column (2), the author replaces industry fixed effects with firm fixed effects. The definitions of the control variables are provided in Appendix A. The standard errors are clustered at the firm level. Statistical significance at the 1, 5, and 10% levels is indicated by ***, **, and *, respectively. Bold text denotes the key variables and their estimated coefficients.

## Cross-sectional analyses

In Section Cross-sectional analyses, cross-sectional analyses are conducted to offer additional evidence on the main hypothesis. H1a suggests that if a firm discloses more non-standardized numerical information about its ESG performance, agencies are more likely to use their own expertise and put their personalized interpretations and judgments on these pieces of information, thereby leading to more rating disagreement. Therefore, the author predicts that numerical disclosure of firms that concentrate on a specific industry would generate more disagreement. Compared with diversified companies, those companies may disclose more industry-specific ESG information, which exacerbates disagreement among raters. Taking SynTao Green Finance for example, its evaluation system consists of general indicators and industry-specific indicators. General indicators are relatively easy to agree among raters, where as industry-specific indicators depend entirely on the rating agency's own expertise and therefore vary widely among raters.

The author uses the variable *Diversification*, defined as the Herfindahl Index of operating income in a company (the quadratic sum of the ratio between income of business k and total income), to divide the full sample into diversified business companies (high-*Diversification* group) and single business companies (low-*Diversification* group). The columns (1) and (2) in Table 7 report the regression results for diversified business companies and single business companies, respectively. The coefficients of *ESG_Qmetrics* are 0.051 in the diversified business group and 0.144 in the single business group, and the difference of the two coefficients is significant at the 5% level. Consistent with the conjecture of the author, non-standardized numerical ESG disclosure causes greater rating divergence in single business companies rather than in diversified business companies.

**Table 7:** Heterogeneous effects of ESG quantitative disclosure on ESG ratings divergence: role of diversified operation and ESG performances.

**Dependent variable: *ESG_Divergence***	***Diversification***		***ESG_mean***	
	**High**	**Low**	**High**	**Low**
	**(1)**	**(2)**	**(3)**	**(4)**
***ESG_Qmetrics***	0.051*	0.144***	**0.025**	0.087***
	**(1.95)**	**(4.99)**	**(1.38)**	**(3.32)**
**Diff. (*****p*****-value) in** ***ESG_Qmetrics***	**0.041**	**0.088**
*ESG _mean*	−0.067**	−0.103***		
	(−2.29)	(−3.43)		
*ESG_N*	0.335***	0.178***	0.438***	0.032
	(5.55)	(3.01)	(10.13)	(0.44)
*State*	−0.011	0.300*	−0.126	0.257**
	(−0.07)	(1.85)	(−0.92)	(2.47)
*Size*	0.182***	0.147**	0.044	0.198***
	(2.97)	(2.09)	(0.92)	(3.86)
*Leverage*	−0.299	−0.075	−0.110	−0.486***
	(−1.35)	(−0.31)	(−0.60)	(−2.62)
*BM*	−0.283*	−0.473***	−0.086	−0.333**
	(−1.72)	(−2.80)	(−0.68)	(−2.40)
*TobinQ*	−0.022	−0.000	0.009	−0.034
	(−0.64)	(−0.01)	(0.43)	(−1.58)
*ROA*	0.582	0.075	0.174	0.570**
	(1.60)	(0.19)	(0.51)	(2.08)
Constant	−1.810	−0.657	0.823	−2.371**
	(−1.32)	(−0.41)	(0.76)	(−2.08)
Year FE	Yes	Yes	Yes	Yes
Agency FE	Yes	Yes	Yes	Yes
Firm FE	Yes	Yes	Yes	Yes
Observations	1,933	1,926	2,487	2,479
R2	0.491	0.506	0.512	0.411

This table presents the regression results of cross-sectional analyses based on Equation (1). Columns (1) and (2) report the estimations of high-Diversification group and low-Diversification group, and columns (3) and (4) report the estimations of high-ESG_mean group and low- ESG_mean group. Variable Diversification is defined as the Herfindahl Index of operating income in a company (the quadratic sum of the ratio between income of business k and total income). The definitions of other variables are provided in Appendix A. The standard errors are clustered at the firm level. The values of t-statistics are in parentheses. Statistical significance at the 1, 5, and 10% levels is indicated by ***, **, and *, respectively. Bold text denotes the key variables and their estimated coefficients.

Next, the author examines whether the rating dispersion stemming from numerical ESG information is heterogenous among firms with different ESG performances. Rating agencies are easier to reach an agreement on positive ESG events rather than negative ESG events, especially if the overall level of ESG performance is poor. Different rating agencies are likely to identify the same negative ESG event of a firm to be different levels of severity. For instance, CASVI will evaluate a firm with a major violation of laws or regulations as “D” level (the lowest level) directly, while Rankins may lower the grade to some extent. Based on variable *ESG_mean*, the author partitions the full sample into two subgroups: firms with better ESG performance than average (high-*ESG_mean* group) and firms with poorer ESG performance than average (low-*ESG_mean* group). The columns (3) and (4) in Table 7 show the estimation results. As predicted, the coefficients of *ESG_Qmetrics* are 0.025 in the better ESG performance group and 0.087 in the poorer ESG performance group, respectively. The difference between the two coefficients is significant, suggesting that agencies tend to disagree more on the quantified ESG disclosure of firms with poor ESG performance.

## Robustness test

The author performs a number of robustness tests in this section. First of all, Equation (1) is re-estimated with alternative measures of ESG rating divergence. In the previous tests, the author has employed *ESG_Divergence*, defined as the standard deviation of ESG ratings of firm *i* in year *t*, to proxy for ESG rating disagreement. Following prior literature (Christensen et al., 2022), the author constructed the alternative variable *Divergence1* as the average of absolute values of the difference between pairs of ratings that firm *I* receives from rating agencies for its ESG performance in year *t*, and *Divergence2* as the coefficient of variation of firm *i*'s ESG ratings in year *t*. In Table 8, columns (1) and (2) show the regression results using alternative dependent variables. The coefficients of *ESG_Qmetrics* are still significantly positive, indicating that the main finding is robust.

**Table 8:** Robustness checks: using alternative measures, alternative sample, and alternative specifications.

**Dependent variable**	**(1)**	**(2)**	**(3)**	**(4)**	**(5)**
	***Divergence1***	***Divergence2***	***ESG_Divergence***	***ESG_Divergence***	***ESG_Divergence***
***ESG_Qmetrics***	0.075***	0.009***	0.062***	0.061**	0.050***
	**(2.88)**	**(2.63)**	**(3.26)**	**(2.51)**	**(2.59)**
*ESG _mean*	−0.047	−0.073***	−0.095***	−0.063	−0.074**
	(−0.98)	(-11.55)	(−2.99)	(−1.53)	(−2.10)
*ESG_N*	0.380***	0.053***	0.410***	0.393***	0.390***
	(5.10)	(5.75)	(8.06)	(4.23)	(7.46)
*State*	0.176	0.029	0.078	0.116	0.130
	(1.36)	(1.50)	(0.78)	(1.29)	(1.32)
*Size*	0.199***	0.032***	0.177***	0.159**	0.124***
	(3.07)	(3.74)	(4.05)	(2.66)	(2.63)
*Leverage*	−0.367*	−0.066**	−0.317**	−0.336*	−0.321**
	(−1.66)	(−2.14)	(−2.00)	(−2.04)	(−1.98)
*BM*	−0.357**	−0.049**	−0.273***	−0.289**	−0.240**
	(−2.49)	(−2.56)	(−2.58)	(−3.29)	(−2.23)
*TobinQ*	−0.000	0.000	0.002	−0.004	−0.005
	(−0.02)	(0.06)	(0.14)	(−0.25)	(−0.26)
*ROA*	0.550	0.047	0.442	0.365	0.376
	(1.53)	(0.96)	(1.60)	(1.16)	(1.43)
*Verification*					−0.051
					(−0.63)
*Willing*					0.226***
					(5.19)
*Inst*					−0.001
					(−0.53)
Constant	−1.386	0.030	−1.675*	−1.798	−0.682
	(−1.00)	(0.16)	(−1.76)	(−1.46)	(−0.67)
Year FE	Yes	Yes	Yes	Yes	Yes
Agency FE	Yes	Yes	Yes	Yes	Yes
Firm FE	Yes	Yes	Yes	Yes	Yes
Observations	4,966	4,966	5,389	4,936	4,961
R2	0.428	0.645	0.459	0.759	0.493

This table presents a battery of robustness checks. Columns (1) and (2) report the regression results using alternative dependent variables (Divergence1 and Divergence2), where Divergence1 is measured as the average of absolute values of the difference between pairs of ratings that firm i receives from rating agencies for its ESG performance in year t, and Divergence2 is measured as the coefficient of variation of firm i's ESG ratings in year t. Column (3) presents the results when the sample extends to all sectors, and column (4) presents the results from the estimation of Equation (1) by clustering firm and year. Column (5) reports the estimation of the results after controlling for a few additional control variables. The definitions of these variables are provided in Appendix A. The standard errors are clustered at the firm level. The values of t-statistics are in parentheses. Statistical significance at the 1, 5, and 10% levels is indicated by ***, **, and *, respectively. Bold text denotes the key variables and their estimated coefficients.

Next, the main analysis is repeated using a redefined sample. Earlier in the article, the author used the sample excluding observations operating in financial service industry on account of intrinsic differences between financial and other sectors companies. In this test, the author extended the sample for regression estimation to all industries. In Table 8, column (3) reports that the coefficient of *ESG_Qmetrics* is 0.062 and significant at the 1% level, providing robust evidence in favor of the hypothesis of author. Additionally, the untabulated results of the DID model (in Section Standardized quantitative ESG disclosure and ESG ratings divergence) and Equation (3) (in Section Consequences: ESG rating in future) also corroborate the earlier findings.

There may be correlations between a given individual's model errors in different periods in a panel data (Colin Cameron and Miller, 2015). To mitigate the potential heteroscedasticity and sequence-related problems, the author replicates the main test by two-way clustering (both at firm and year levels) and the result in Table 8, column (4), is similar. The coefficient of *ESG_Qmetrics* remains significantly positive and, again, supports the conclusions of the author.

In the fourth robustness test, the author controls for potential omitted factors that affect ESG rating divergence. Although firm fixed effects and year fixed effects are controlled, unobservable factors may affect rating divergence differently. First, the author controls for whether the ESG report or CSR report is verified by a third party (*Verification*). Southworth (2009) studied corporate voluntary action as a mechanism for addressing the related problems of climate change and energy security and found that third-party verification of emission data contributes to valuable comparisons across industries and companies. Accordingly, it is reasonable to speculate that third-party verification of a firm's ESG disclosure would, to some extent, narrow rating differences and trigger convergence of agencies' ratings. The author constructs an indicator variable, *Verification*, defined as 1 if the firm's ESG report or CSR report is verified by a third party and 0 otherwise. Second, the author controls for voluntary or mandatory ESG disclosure (*Willing*). In December 2008, the Shanghai and Shenzhen Stock Exchange announced regulations that obligated a subset of listed companies to file CSR reports along with their annual reports. Rating agencies may conduct an assessment of mandatory ESG disclosure and voluntary ESG disclosure differently. *Willing* is a dummy variable that is equal to 1 if a firm discloses ESG/CSR report under the requirements and zero otherwise. Finally, the author further controls for the effect of institutional ownership (*Inst*). Institutional investors play a prominent role in shaping ESG performance, and firms with greater institutional ownership tend to have lower ESG rating divergence (Dyck et al., 2019; Kim et al., 2019; Christensen et al., 2022). Following previous studies, *Inst* is measured as the percentage of the firm's shares owned by institutional investors. In Table 8, column (5) reports the regression result after controlling *Verification, Willing*, and *Inst*. The coefficient of *ESG_Qmetrics* is positive and significant at the 1% level after controlling these variables, confirming the main conclusion.

## Discussion

Using relevant data of the Shanghai and Shenzhen A-share non-financial listed companies from 2014 to 2020, this study examines whether quantitative ESG disclosure is conducive to rating convergence among agencies. In particular, to include as many raters as possible, the author uses Chinese ESG rating data provided by six ESG rating agencies, which represent the major players in Chinese ESG rating market: SynTao Green Finance, Sino-Securities Index, CASVI, WIND ESG, FTSE Russell, and Rankins. The empirical results show that the divergence of ESG ratings is significantly positive with non-standardized numerical ESG disclosure, especially with disclosure on environmental and social issues. This relationship remains unchanged after a series of robustness tests. To mitigate the potential endogenous problem and examine whether standardized quantitative disclosure helps to reduce rating disagreement, the author employs the quasi-natural experiment brought by the implementation of Environmental, Social and Governance Reporting Guide introduced by Hong Kong Exchanges (HKEX) and conduct a DID estimation. The results suggest that if ESG disclosure is standardized and comparable, more numerical information reduces agencies' rating disagreement instead. Additionally, the lack of agreement is associated with a low rating in the future, indicating that disagreement represents a source of uncertainty on potential risks. Further analyses show that the impact of quantified ESG disclosure on rating disagreement is more pronounced when firms develop a single business rather than a diversified business and experience poor ESG performance rather than good ESG performance.

This article draws on Christensen et al. (2022)'s research methodology, but there are several differences in the main identification model that are noteworthy. First, they use ESG disclosure scores provided by Bloomberg to capture the level of firms' ESG disclosure, ranging from 0.1 to 100. The disclosure score is higher if a company discloses more data points that Bloomberg collects. In this study, the author focuses on quantitative disclosure rather than overall disclosure. Therefore, the author uses the data of quantified indicators about firms' ESG disclosure provided by the CSMAR database. CSMAR extracts substantial and quantified metrics related to environmental, social, and governance activities and performances by scanning through firms' CSR/ESG reports. Depending on these data points, the variable *ESG_Qmetrics* counts the number of quantified indicators about firms' ESG disclosure. The difference between the research questions determines that the author uses the different data and variable to measure the ESG disclosure than they did. Second, Christensen et al. (2022) obtained ESG ratings from three agencies, namely, Morgan Stanley Capital International's IntangibleValue Assessment, Thomson Reuters' ASSET4, and Sustainalytics, because these agencies publicly released ESG ratings and provided international data that covered their sample. In this study, the sample consists of Chinese A-share public firms, so the rating data available changed. The author uses ESG ratings provided by six raters, SynTao Green Finance, Sino-Securities Index, CASVI, WIND ESG, FTSE Russell, and Rankins, who together represent the major players in Chinese ESG rating space. Since rating divergence is the interest, we both require observations to have ratings from more than one rater. It means that the number of ratings of a firm-year in Christensen et al. (2022) is 2 or 3, while the number ranges from 2 to 6 in this study. Compared with them, the author adds the number of ratings available to control variables in order to control for their effect on rating disagreement. Third, because of the higher potential influence of the number of ratings in this study than that in Christensen et al. (2022), the author conducts some tests that re-estimate the main model when the number of ratings is the same or close. The untabulated results show that the effect of quantitative disclosure on rating divergence does not change.

This study provides further empirical evidence of the presence and determinants of rating disagreement. If raters adopt the same approach to assess firms' ESG performance, rating divergence could come only from different information sets such as raters' private information about firms. In this context, greater public disclosure, which means less private information, would lead to higher rating consistency. However, the empirical evidence in this study documents a strong positive relationship between quantitative ESG disclosure and divergence of ESG ratings, revealing that rating agencies use their own expertise and put their personalized interpretations and judgments on firms' disclosure. This finding is supported by previous studies (Chatterji et al., 2016; Berg et al., 2022). Chatterji et al. (2016) demonstrate convincingly that raters apply diverse theories of social responsibility, and select various topics (lack of common theorization) and metrics (lack of commensurability) in their evaluation framework. Berg et al. (2022) find that rating disagreement stems from different sets of attributes (scope divergence), different indicators measuring the same attribute (measurement divergence), and different views on the relative importance of the attributes (weights divergence).

The DID tests (Section Standardized quantitative ESG disclosure and ESG ratings divergence) and cross-sectional analyses (Section Cross-sectional analyses) in this article add further evidence that the lack of common norms for interpreting ESG disclosure brings about the lack of agreement across the information intermediaries. Employing the quasi-natural experiment brought by HKEX, this study finds that despite more quantitative disclosures after the new guidance, the divergence has been alleviated significantly, because standardized numerical disclosures required by the guidance are more comprehensible and comparable. Next, the regression results show that the numerical disclosure of firms with a single business rather than a diversified business generates more disagreement. Taking SynTao Green Finance for instance, its evaluation system consists of general and industry-specific indicators. General indicators are appropriate for all companies and generally regarded as relatively established fields with little controversy, while industry-specific indicators are more complex and entirely dependent on agencies' industry expertise and thus vary widely. Firms developing a single business are more likely to disclose information with industry specialization and therefore undertake more rating divergence. In addition, the author finds that agencies tend to disagree more on the quantified ESG disclosure of firms with poor ESG performance rather than good ESG performance. There could be two circumstances with a poor ESG performance firm: low degree of disclosure and negative ESG events. Although no disclosure almost leads to a general consensus, negative events are perceived as different levels of severity. For instance, CASVI will evaluate a firm with a major violation of laws or regulations as “D” level (the lowest level) directly, while Rankins may lower the grade to some extent. Overall, these results all validate H1a that quantitative ESG disclosure is positively related to divergence of ESG ratings because of different interpretations and judgments of information.

## Conclusions

The use of ESG ratings in academic research and investment practice has continued to accelerate recently. In the meantime, the issue of ESG rating disagreement has also spurred a considerable concern in the financial press, investors, and researchers. Obviously, it is warranted to examine the validity of ESG ratings and explore the dynamics driving convergence among information intermediaries.

Using rating data from six ESG rating providers in China, the author examines whether quantitative ESG disclosure is conducive to rating convergence among agencies. The results show that greater quantified ESG disclosure brings about greater divergence of ESG ratings. Specifically, quantitative disclosure on environmental and social issues plays a greater role compared with governance disclosure in explaining rating disagreement.

To mitigate the potential endogenous problem and examine whether standardized quantitative disclosure helps to reduce the rating disagreement, the author uses a shock of the implementation of Environmental, Social and Governance Reporting Guide introduced by Hong Kong Exchanges (HKEX) and conducts a DID estimation. The estimation results support the conjecture. In addition, further analyses show that the lack of agreement is associated with a low rating in the future, indicating that disagreement represents a source of uncertainty on potential risks. The author also finds that the effect of quantified ESG disclosure on rating dive is more significant when firms are single businesses rather than diversified businesses with poor ESG performance rather than good ESG performance. Lastly, the main results are robust to alternative measures of ESG rating divergence, alternative sample, two-way clustering, and additional control variables.

This study responds to the heated debate on ESG rating divergence in the following aspects. First of all, it enriches the academic evidence of ESG rating disagreement. It should be noted that this study is related to Christensen et al. (2022) with the conclusion that rating divergence is larger when firms disclose more. Specifically, this study differs from theirs in that the author explores how quantitative disclosure influences the evaluation results of rating agencies about ESG issues. Next, this study complements the literature related to sociology of evaluation. Although quantification enhances comprehensibility and comparability (Porter, 1995; Espeland and Stevens, 1998, 2008), rating disagreement is more pronounced when firms disclose more non-standardized numerical information, indicating that low commensurability still poses a serious challenge in newly emergent areas. Finally, this study has implications for research on economic consequences of ESG rating disagreement. Gibson Brandon et al. (2021) and Avramov et al. (2022) find that firms with high ESG rating disagreement require an equity premium because disagreement is perceived as a source of uncertainty. This study complements some evidence and demonstrates that divergence is associated with a lower average rating in the future, indicating that there would be potential risks with those firms.

This study not only contributes to the theoretical debate on why ESG raters disagree but also has important implications in practice. Researchers should carefully assess and explain data analyses based on ESG ratings, especially if using one particular agency's rating. If possible, use multiple measures of ratings to proxy ESG performance as robustness tests to enhance the confidence of these academic studies. What is more, this study also calls for closer cooperation among companies, rating agencies, and policymakers to build a consensus on crucial ESG performance and establish consistent and transparent disclosure standards. Meanwhile, rating agencies should become more transparent about their rating methodologies and valuation systems. Improvement in transparency will help rating users to locate the source of rating disagreement and evaluate whether the disagreement affects their decisions.

This study has several limitations that should be noted. First, this study uses the number of materials and quantified indicators in the ESG or CSR report to measure the degree of a firm's ESG disclosure. As such, future studies may take account of disclosure from other sources, including company websites, news reports, and penalty information from regulatory agencies. Second, restricted to the relatively small dataset, the study focuses on ESG rating divergence in all non-financial industries. Future research could examine the industry heterogeneity of ESG disagreement, i.e., the tobacco, mining, and weapons industries are worthy of attention.

## Data availability statement

The original contributions presented in the study are included in the article/supplementary material, further inquiries can be directed to the corresponding author/s.

## Author contributions

The author confirms being the sole contributor of this study and has approved it for publication.

## Conflict of interest

The author declares that the research was conducted in the absence of any commercial or financial relationships that could be construed as a potential conflict of interest.

## Publisher's note

All claims expressed in this article are solely those of the authors and do not necessarily represent those of their affiliated organizations, or those of the publisher, the editors and the reviewers. Any product that may be evaluated in this article, or claim that may be made by its manufacturer, is not guaranteed or endorsed by the publisher.

## Footnotes

^1^Commensuration is an important concept in sociology of valuation and evaluation. Generally, most quantification can be understood as commensuration (Espeland and Stevens, 2008). In the context of this article, the author does not make a distinction between “quantification” and “commensuration” and uses the term “quantification”.

## Appendix

**Table A:** Variable definitions.

**Variable**	**Definition**
**Panel A: ESG variables**	
ESG_Divergence	The standard deviation of ESG ratings of firm i in year t.
ESG_Qmetrics	The natural logarithm of the number of quantified indicators about firm i's ESG disclosureplus one.
ESG_Qmetrics_E	The natural logarithm of the number of quantified indicators for environmental pillar about firm i's ESG disclosure plus one.
ESG_Qmetrics_S	The natural logarithm of the number of quantified indicators for social pillar about firm i's ESG disclosure plus one.
ESG_Qmetrics_G	The natural logarithm of the number of quantified indicators for governance pillar about firm i's ESG disclosure plus one.
ESG_mean	The average ESG rating of a firm received from different rating agencies.
ESG_N	The number of rating agencies following the firm.
Divergence1	The average of absolute values of the difference between pairs of ratings that firm i receives from rating agencies for its ESG performance in year t.
Divergence2	The coefficient of variation of firm i's ESG ratings in year t.
**Panel B: Fundamental variables**	
State	A dummy variable equal to one if the company is a state-owned company zero otherwise.
Size	The natural logarithm of total assets.
Leverage	The ratio between total debt the book value of assets.
ROA	The ratio of net profit to total assets.
BM	The ratio between book value market value.
TobinQ	Tobin's Q.
Diversification	The quadratic sum of the ratio between income of businessk total income.
Verification	A dummy variable equal to one if the firm's ESG report or CSR report is verified by a third party zero otherwise.
Willing	A dummy variable equal one if the firm discloses ESG/CSR report under the requirements zero otherwise.
Inst	The percentage of the firm's shares owned by institutional investors.

## References

[B1] Akins B (2018). Financial reporting quality and uncertainty about credit risk among ratings agencies. Account. Rev. 93, 1–22. 10.2308/accr-51944

[B2] Avramov D. Cheng S. Lioui A. Tarelli A. (2022). Sustainable investing with ESG rating uncertainty. J. Finan. Econ. 145, 642–664 10.1016/j.jfineco.2021.09.009

[B3] Berg F. Koelbel J. F. Rigobon R. (2022). Aggregate confusion: the divergence of ESG rating. Rev. Financ. 26, 1–30. 10.1093/rof/rfac033

[B4] Billio M. Costola M. Hristova I. Latino C. Pelizzon L. (2021). Inside the ESG ratings:(Dis) agreement and performance. Corp. Soc. Resp. Env. Ma. 28, 1426–1445. 10.1002/csr.2177

[B5] Bonsall S. B. Miller B. P. (2017). The impact of narrative disclosure readability on bond ratings and the cost of debt. *Rev*. Acc. Stud. 22, 608–643. 10.1007/s11142-017-9388-0

[B6] Broadstock D. C. Chan K. Cheng L. T. W. Wang X. (2021). The role of ESG performance during times of financial crisis: evidence from COVID-19 in China. Financ. Res. Lett. 38, 101716. 10.1016/j.frl.2020.101716 32837385 PMC7425769

[B7] Camilleri M. A (2015). Environmental, social and governance disclosures in Europe.Sustain. Account. Mana. 6, 224–242. 10.1108/SAMPJ-10-2014-0065

[B8] Camilleri M. A (2018). Theoretical insights on integrated reporting: the inclusion of non-financial capitals in corporate disclosures. *Corp*. Commun. 23, 567–581. 10.1108/CCIJ-01-2018-0016

[B9] Cao J. Liang H. Zhan X. (2019). Peer effects of corporate social responsibility. Manage. Sci. 65, 5487–5503. 10.1287/mnsc.2018.3100

[B10] Chatterji A. K. Durand R. Levine D. I. Touboul S. (2016). Do ratings of firms converge? Implications for managers, investors and strategy researchers. Strategic. Manage. J. 37, 1597–1614. 10.1002/smj.2407

[B11] Cheng B. Ioannou I. Serafeim G. (2014). Corporate social responsibility and access to finance. Strategic. Manage. J. 35, 1–23. 10.1002/smj.2131

[B12] Christensen D. M. Serafeim G. Sikochi A. (2022). Why is corporate virtue in the eye of the beholder? The case of ESG ratings. Account. Rev. 97, 147–175. 10.2308/TAR-2019-0506

[B13] Colin Cameron A. Miller D. L. (2015). A practitioner's guide to cluster-robust inference. J. Human Res. 50, 317–372. 10.3368/jhr.50.2.317

[B14] Cookson J. A. Niessner M. (2020). Why don't we agree? Evidence from a social network of investors. J. Finance. 75, 173–228. 10.1111/jofi.12852

[B15] Durand R. Rao H. Monin P. (2007). Code and conduct in French cuisine: Impact of code changes on external evaluations. Strategic. Manage. J. 28, 455–472. 10.1002/smj.583

[B16] Dyck A. Lins K. V. Roth L. Wagner H. F. (2019). Do institutional investors drive corporate social responsibility? International evidence. *J. Finan*. Econ. 131, 693–714. 10.1016/j.jfineco.2018.08.013

[B17] Engle R. F. Giglio S. Kelly B. Lee H. Stroebel J. (2020). Hedging climate change news. Rev. Financ. Stud. 33, 1184–1216. 10.1093/rfs/hhz072

[B18] Escrig-Olmedo E. Fernández-Izquierdo M. Á. Ferrero-Ferrero I. Rivera-Lirio J. M. Muñoz-Torres M. J. (2019). Rating the raters: evaluating how ESG rating agencies integrate sustainability principles. Sustain. Basel. 11, 915. 10.3390/su11030915

[B19] Espeland W. N. Sauder M. (2007). Rankings and reactivity: How public measures recreate social worlds. Am. J. Sociol. 113, 1–40. 10.1086/517897

[B20] Espeland W. N. Stevens M. L. (1998). Commensuration as a social process. Annu. Rev. Sociol. 24, 313–343. 10.1146/annurev.soc.24.1.313

[B21] Espeland W. N. Stevens M. L. (2008). A sociology of quantification. *Arch. Eur*. Sociol. 49, 401–436. 10.1017/S000397560900015024085610

[B22] Gibson Brandon R. Krueger P. Schmidt P. S. (2021). ESG rating disagreement and stock returns. Financ. Anal. J. 77, 104–127. 10.1080/0015198X.2021.1963186

[B23] Grewal J. Serafeim G. (2020). Research on corporate sustainability: review and directions for future research. Found. Trends. Account. 14, 73–127. 10.1561/1400000061

[B24] GSIR. (2020). Global Sustainable Investment Review 2020. Global Sustainable Investment Alliance. Available online at: www.gsi-alliance.org/wp-content/uploads/2021/08/GSIR-20201.pdf

[B25] Hartzmark S. M. Sussman A. B. (2019). Do investors value sustainability? A natural experiment examining ranking and fund flows. J. Finance. 74, 2789–2837. 10.1111/jofi.12841

[B26] Hong H. Stein J. C. (2007). Disagreement and the stock market. J. Econ. Perspect. 21, 109–128. 10.1257/jep.21.2.109

[B27] Hope O-. K (2003). Accounting policy disclosures and analysts' forecasts. Contemp. Account. Res. 20, 295–321. 10.1506/LA87-D1NF-BF06-FW1B

[B28] Hsu G. Roberts P. W. Swaminathan A. (2012). Evaluative schemas and the mediating role of critics. Organ. Sci. 23, 83–97. 10.1287/orsc.1100.0630

[B29] Hubbard T. D. Christensen D. M. Graffin S. D. (2017). Higher highs and lower lows: the role of corporate social responsibility in CEO dismissal. Strategic. Manage. J. 38, 2255–2265. 10.1002/smj.2646

[B30] Jørgensen E. N. J. Ellingsen T. H. (2021). ESG Disagreement: Determining Factors and Impact on Stock performance (Handelshøyskolen BI).

[B31] Khan M. Serafeim G. Yoon A. (2016). Corporate sustainability: first evidence on materiality. Account. Rev. 91, 1697–1724. 10.2308/accr-51383

[B32] Kim I. Wan H. Wang B. Yang T. (2019). Institutional investors and corporate environmental, social, and governance policies: evidence from toxics release data. Manage. Sci. 65, 4901–4926. 10.1287/mnsc.2018.3055

[B33] Kotsantonis S. Serafeim G. (2019). Four things no one will tell you about ESG data. J. Appl. Corp. Financ. 31, 50–58. 10.1111/jacf.12346

[B34] Krueger P. Sautner Z. Starks L. T. (2020). The importance of climate risks for institutional investors. Rev. Financ. Stud. 33, 1067–1111. 10.1093/rfs/hhz137

[B35] Lins K. V. Servaes H. Tamayo A. N. E. (2017). Social capital, trust, and firm performance: the value of corporate social responsibility during the financial crisis. J. Fin. 72, 1785–1824. 10.1111/jofi.12505

[B36] Morgan D. P (2002). Rating banks: Risk and uncertainty in an opaque industry. Amer. Econ. Rev. 92, 874–888. 10.1257/00028280260344506

[B37] Pedersen L. H. Fitzgibbons S. Pomorski L. (2021). Responsible investing: the ESG-efficient frontier. J. Finan. Econ. 142, 572–597. 10.1016/j.jfineco.2020.11.001

[B38] Porter T. M (1995). Trust in Numbers Princeton, NJ: Princeton University Press.

[B39] Sauder M. Espeland W. N. (2009). The discipline of rankings: tight coupling and organizational change. Am. Sociol. Rev. 74, 63–82. 10.1177/000312240907400104

[B40] Servaes H. Tamayo A. (2013). The impact of corporate social responsibility on firm value: The role of customer awareness. Manage. Sci. 59, 1045–1061. 10.1287/mnsc.1120.1630

[B41] Southworth K (2009). Corporate voluntary action: A valuable but incomplete solution to climate change and energy security challenges. Policy. Soc. 27, 329–350. 10.1016/j.polsoc.2009.01.008

[B42] Tang D. Y. Yan J. Yao C. Y. (2021). The Determinants of ESG Ratings: Rater Ownership Matters. Working paper.

